# Unmasking of Constrictive Pericarditis Ventricular Interdependence After Transcatheter Aortic Valve Replacement

**DOI:** 10.1016/j.jscai.2022.100512

**Published:** 2022-10-19

**Authors:** Ahmad Mustafa, Sherif Elhosseiny, Craig Basman, Gregory Maniatis, Chad Kliger, Andrew Warchol, Thinzer Shwe, Arber Kodra, Mitchell D. Weinberg, James Lafferty

**Affiliations:** a Department of Cardiology, Staten Island University Hospital, Staten Island, New York; b Department of Cardiology, Lenox Hill Hospital, New York, New York

**Keywords:** constrictive pericarditis, transcatheter aortic valve replacement

A 59-year-old man presented with dyspnea on exertion and bilateral lower-extremity swelling over the previous 3 months. His medical history included Hodgkin lymphoma, for which he had received chemotherapy and radiation therapy 21 years ago. Physical examination was significant for basal crackles on lung auscultation, a jugular venous pressure of 15 cm, ascites, 3+ bilateral lower-extremity swelling, and a grade 5 or 6 systolic ejection murmur with a soft second heart sound.

Electrocardiogram showed a normal sinus rhythm with no conduction abnormalities. Echocardiogram revealed an ejection fraction of 60% along with severe aortic stenosis (AS), with an aortic valve area of 0.90 cm^2^ and a mean gradient of 44 mm Hg (Figure 1A). Moreover, thickening/calcification of the pericardium was noted on the echocardiogram. Computed tomography of the chest showed moderate bilateral pleural effusions along with extensive pericardial calcifications and thickening (Figure 1B); however, transthoracic echocardiography did not reveal a shift in inflow velocities and/or an interventricular septal “bounce” with respiratory variation that would be diagnostic of constrictive pericarditis (CP). Cardiac catheterization showed nonobstructive coronary artery disease. Invasive hemodynamic tracings showed prominent Y descent and a “dip and plateau” sign; however, simultaneous right ventricular and left ventricular pressure tracings did not reveal ventricular interdependence (Figure 1C).

**Figure 1:**
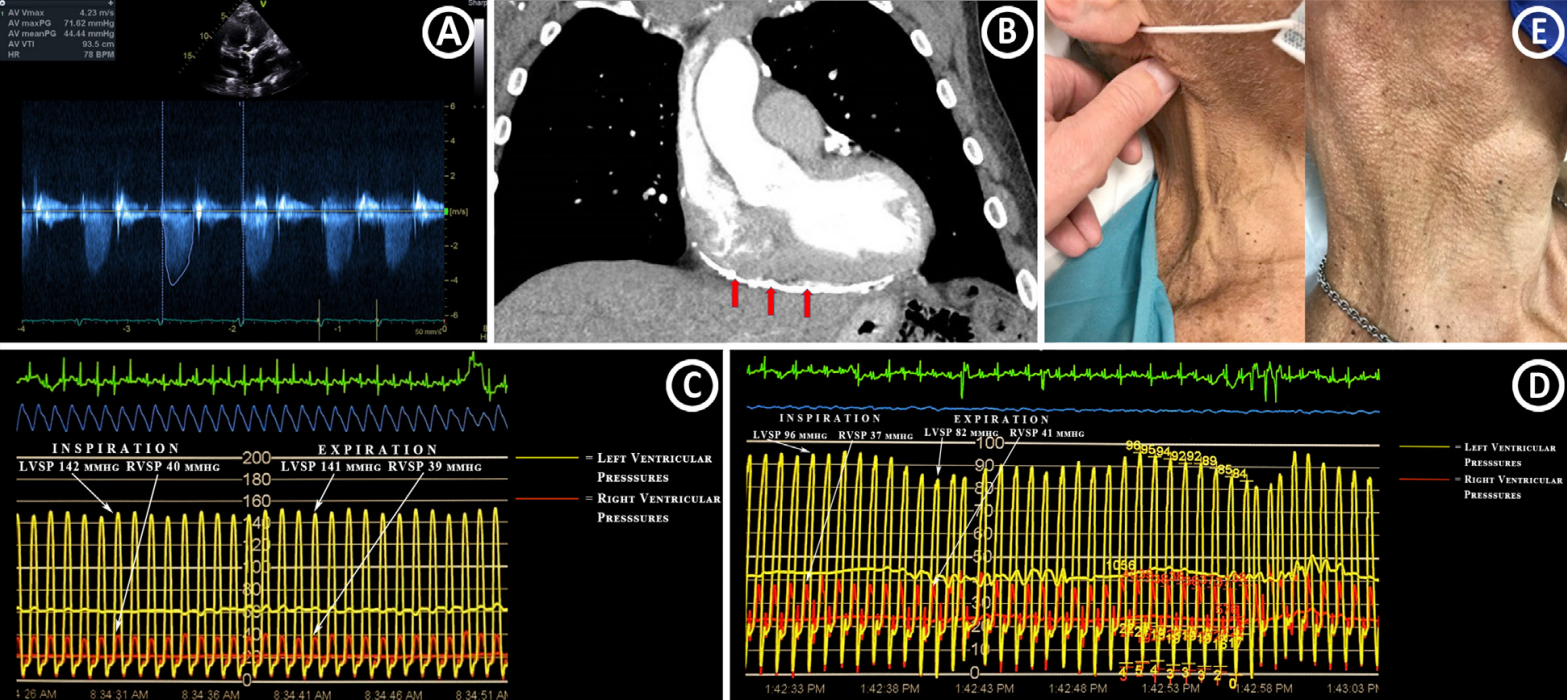
(**A**) Severe aortic stenosis with a mean gradient of 44 mm Hg on an echocardiogram. (**B**) A computed tomography scan showing pericardial calcification and thickening (red arrows). (**C**) Invasive hemodynamic tracing before transcatheter aortic valve replacement (TAVR) showing the absence of ventricular interdependence. (**D**) Post-TAVR invasive hemodynamic tracing showing the presence (unmasking) of ventricular interdependence. During inspiration, the peak left ventricular systolic pressure is (LVSP) reduced, with a corresponding increase in the right ventricular systolic pressure (RVSP). During expiration, the peak LVSP increased, with a corresponding decrease in the RVSP. (**E**) Before (left) and after (right) TAVR and pericardiectomy.

Owing to a strong suspicion of CP along with severe AS, surgical consultation was obtained; however, the patient was deemed a high-risk candidate for both surgical aortic valve replacement and pericardiectomy. Hence, transcatheter aortic valve replacement (TAVR) was scheduled, followed by a staged pericardiectomy. After optimization of fluid status with aggressive diuresis, the patient underwent an uncomplicated TAVR with a balloon-expandable transcatheter heart valve. He then underwent a repeat cardiac catheterization before the planned staged pericardiectomy. After TAVR, the ventricular interdependence was “unmasked,” with right-to-left interventricular pressure discordance along with a “square root” sign (Figure 1D). The patient later underwent pericardiectomy, which resulted in a marked reduction of heart failure symptoms (Supplemental Video 1)

The postoperative course after pericardiectomy was uncomplicated. He reported a marked reduction in clinical symptoms and reduced need for diuretic therapy. He was back to his usual exercise capacity and denied any symptoms of dyspnea or lower-extremity swelling (Figure 1E).

## Discussion

Radiation-induced heart disease is a side effect of radiation therapy and includes restrictive cardiomyopathy, pericardial diseases, coronary artery disease, conduction system abnormalities, and valvular heart disease. In patients with prior mediastinal radiation exposure, AS and CP may coexist, and this combination has been associated with long-term mortality.^1^^,^^2^ The diagnosis of CP in such cases can be challenging, and a high level of clinical suspicion is required.^3^ Diagnosis is based mainly on 1 or more imaging modalities, such as echocardiography, computed tomography scan, cardiac magnetic resonance imaging, and hemodynamic assessment during cardiac catheterization.^4^^,^^5^ Classic cardiac catheterization findings include the dip and plateau or square root sign, diastolic pressure equalization, and ventricular interdependence. In patients with severe AS, an increase in the left ventricular afterload may prevent leftward septal bowing during inspiration. Accordingly, “masking” of the ventricular interdependence may occur, complicating the diagnosis of CP with severe AS. In our case, TAVR reduced the afterload, which ultimately led to the unmasking of ventricular interdependence.

**Constriction/restriction pearls**

1. Interpreting the dip and plateau not only depends on the fidelity of the system (underdamped = dip and plateau) but also has a poor clinical specificity.
2. Ventricular interdependence is the hallmark of constriction and requires equal fidelity of both catheter systems. For example, a standard pulmonary artery catheter does not have the same fidelity as a pigtail catheter. Consider using 2 pigtail catheters.
3. Appropriate hemodynamics require a regular rhythm. For patients with atrial fibrillation, use pacing during the assessment.
4. Remember that the classic hemodynamics of constriction were not determined in patients with confounding variables such as AS. A high index of suspicion for CP is required to make the diagnosis.

## Supplementary material

Video 1 Thick calcified pericardium during pericardiectomy.
